# Using *Wolbachia* Releases to Estimate *Aedes aegypti* (Diptera: Culicidae) Population Size and Survival

**DOI:** 10.1371/journal.pone.0160196

**Published:** 2016-08-01

**Authors:** Gabriela de Azambuja Garcia, Lilha Maria Barbosa dos Santos, Daniel Antunes Maciel Villela, Rafael Maciel-de-Freitas

**Affiliations:** 1 Laboratório de Transmissão de Hematozoários, Instituto Oswaldo Cruz, Fundação Oswaldo Cruz (IOC/FIOCRUZ), Rio de Janeiro, Brazil; 2 Programa de Computação Científica, Fundação Oswaldo Cruz (PROCC), Rio de Janeiro, Brazil; New Mexico State University, UNITED STATES

## Abstract

Mosquitoes carrying the endosymbiont bacterium *Wolbachia* have been deployed in field trials as a biological control intervention due to *Wolbachia* effects on reducing transmission of arboviruses. We performed mark, release and recapture (MRR) experiments using *Wolbachia* as an internal marker with daily collections with BG-Traps during the first two weeks of releases in Rio de Janeiro, Brazil. The MRR design allowed us to investigate two critical parameters that determine whether *Wolbachia* would successful invade a field population: the probability of daily survival (PDS) of *Wolbachia*-infected *Aedes aegypti* females, and the wild population density during releases. Released females had a PDS of 0.82 and 0.89 in the first and second weeks, respectively, immediately after releases, which is well within the range of previous estimates of survivorship of wild mosquitoes in Rio de Janeiro. Abundance estimation of wild population varied up to 10-fold higher depending on the estimation method used (634–3565 females on the average-difference model to 6365–16188 females according to Lincoln-Petersen). *Wolbachia*-released mosquitoes were lower than the density estimation of their wild counterparts, irrespectively of the model used. Individually screening mosquitoes for the presence of *Wolbachia* reduced uncertainty on abundance estimations due to fluctuation in capturing per week. A successful invasion into local population requires *Ae*. *aegypti* fitness is unaffected by *Wolbachia* presence, but also reliable estimates on the population size of wild mosquitoes.

## Introduction

The distribution of diseases such as malaria and dengue frequently overlaps with tropical and subtropical zones in which primary vectors of these diseases are more abundant [1,2]. Considering dengue virus (DENV), about half of the world's population lives at risk of getting infected. In particular, Brazil registered more than one million cases annually in the last three years [3,4]. Since 2010 Brazil has all four DENV serotypes circulating in the country, promoting dengue outbreaks every 4–5 years, often due to the arrival of a new serotype in a susceptible human host population [5–7]. Apart from dengue, two other arboviruses were recently detected in Brazil: chikungunya (CHIKV) [8] and Zika (ZIKV) [9]. The potential association between ZIKV during pregnancy and microcephaly in newborn has raised additional concerns about vector control efforts to mitigate arboviruses transmission. Current evidence shows DENV, CHIKV and ZIKV to be overwhelmingly transmitted by *Aedes* mosquitoes, especially *Aedes aegypti* [10,11], but other mosquitoes in the case of ZIKV [12]. The main role of *Ae*. *aegypti* as vector of these three arboviruses is probably due to its close association with human dwellings, since females lay eggs in man-made containers, bite preferably human hosts and are more abundant in urbanized landscapes with low vegetation coverage [13–16].

Since there is no vaccine currently available, the best way to reduce arboviruses transmission still relies on vector control, which ultimately aims to maintain *Ae*. *aegypti* density below a theoretical threshold to avoid outbreaks [17]. Thus, estimations on mosquito population size, survivorship and spatial distribution in endemic areas becomes critical for improving practices in vector control, e.g., directing the intensification of mechanical and chemical control activities in the districts in which vector population is higher [6, 18, 19].

In Brazil, *Ae*. *aegypti* density is often estimated by indexes derived from infestation rates based on larval surveys, in which a sample of around 10% of houses are randomly selected and inspected 4–6 times yearly [20]. These indexes do not provide good estimators on adult mosquito abundance because container productivity and larval mortality are not taken into account [21,22]. Estimates on adult mosquito population density might be achieved through adult sampling using traps [23,24] and mark, release and recapture (MRR) experiments [25–27]. MRR-based estimation has been proposed as a more reliable approach to determine *Ae*. *aegypti* population size because it focuses on adult sampling, providing more robust estimates on the mosquito life cycle stage directly responsible for disease transmission [28,29].

One of the most promising strategies designed to reduce arboviruses transmission uses the intracellular endosymbiont, maternally inherited bacterium *Wolbachia pipientis*, which is naturally present in up to 65% of all insects [30–32]. This approach explores the fact that *Wolbachia* reduces transmission of key pathogens, including DENV and CHIKV viruses in the *Ae*. *aegypti* mosquitoes [33,34]. The *w*Mel strain causes a phenotypic effect called cytoplasmic incompatibility (CI), which is a reproductive incompatibility that prevents females without *Wolbachia* from producing viable offspring after mating with *Wolbachia*-infected males. By contrast, *Wolbachia*-infected females can successfully reproduce after mating with either *Wolbachia*-infected or wild male [35]. This reproductive advantage increases the frequency of *Wolbachia* infection in a given population with each subsequent generation. Thus, the control strategy is to release mosquitoes with this bacterium for 10 or more consecutive weeks [36]. The expected outcome is the replacement of wild, vector competent, mosquitoes with *Wolbachia*-infected mosquitoes, potentially ameliorating the burden of arboviruses.

In the first two weeks of releases, *Wolbachia* may be seen as an internal marker, allowing estimates on the probability of daily survival rates of released *Wolbachia*-infected *Ae*. *aegypti* females, but also the population size of wild mosquitoes during releases [37]. These estimates are critical for understanding invasion, because: (1) the daily survival rate of *Wolbachia*-released mosquitoes is a strong indicator whether *Ae*. *aegypti* females are fit to field conditions, (2) the displacement of wild vector populations by refractory *Wolbachia*-carrying mosquitoes have been shown to have a threshold conditions dictated by *Wolbachia* frequency in the total population [38]; and (3) to calculate the ideal minimum number of *Wolbachia*-carrying mosquitoes that have to be released per week to succeed invasion promoting low nuisance in local residents. Therefore, our main objective is to estimate the probability of daily survival rates of *Ae*. *aegypti* infected with *Wolbachia* and population density of wild *Ae*. *aegypti* for better characterize *Wolbachia* invasion.

## Materials and Methods

### Study area

We conducted MRR studies in the district of Tubiacanga, Rio de Janeiro (area: 8.6 ha; 22°47′08′′S; 43°13′36′′W). Such locality is an isolated middle-class suburban area located on the offshores of Guanabara Bay and distant 2.1Km for the closest community, which discourages mosquito migration. This residential area has paved streets, well-maintained sidewalks and low-moderate vegetation coverage, with around 2902 people living in 867 houses. Most houses have 2–3 bedrooms, large yards, regular water supply and garbage collection.

### Mosquitoes release

The strain *w*MelBr was derived from the backcrossing of Australian *Ae*. *aegypti* females *w*Mel-infected with Brazilian males (collected from four districts in Rio de Janeiro) during nine generations [39]. Larvae were reared under optimal rearing conditions, in plastic trays in 3 liters of filtered and dechlorinated water (500 larvae per tray), and fed with 0.45g of Tetramin**®** Tropical Flakes Fish Food every day. Adult mosquitoes were maintained in a climate controlled insectary, at 27 ± 1°C and 65 ± 5% RH, with a 12:12 hour light:dark cycle, and received constant 10% sucrose solution up to the release day. Before every release, a sample of 100 mosquitoes was screened for *Wolbachia* to confirm the infection. Releases were conducted outdoors at 05:00AM, once a week, with 5–6 days-old adults at 1:1 sex ratio, with 50 mosquitoes released every four houses. A total of 2,350 *Wolbachia*-carrying females were released in field each week (2.71 per premise). Releases lasted 20 weeks, but our analyzes considered only the first two weeks, when captured mosquitoes positive for *Wolbachia* were those we released.

### Mosquito collection

Mosquitoes were captured using 30 BG-Sentinel**®** Traps (BGS, BioGents, Regensburg, Germany) uniformly distributed across Tubiacanga. The kind of trap consists of a collapsible white bucket with white gauze covering its opening. In the middle of the gauze cover, a black tube through which a down flow is created by a fan that causes any mosquito surrounding the trap to be sucked into a catch bag. BGS-Trap uses local energy power and captures predominantly host-seeking *Ae*. *aegypti* females, seldom males [40,41]. After starting releases, traps were inspected daily for 14 days, and only female counts were included in the analyses.

### Monitoring Wolbachia infection in field-collected mosquitoes

All mosquitoes collected in the BGS-Trap were brought to lab in order to proceed with identification using taxonomic keys [10]. Those classified as *Ae*. *aegypti* were screened for the presence of *Wolbachia*, following the approach taken in Dutra et al 2016 [42].

### Estimating the daily survival of Wolbachia-carrying mosquitoes in field

The daily captures of mosquitoes allowed us to estimate the probability of daily survival (PDS) by using the exponential model [43]. Traditionally, the exponential model has been used to describe mortality patterns in MRR experiments with *Ae*. *aegypti* procedures [15, 28, 44]. This model assumes that marked, captured individual counts vary over time as an exponential decay with a constant rate, i.e., mosquito mortality is independent of age. This assumption is reasonable given typical mosquito life span.

### Estimating the population size of wild mosquitoes using conventional MRR models

The wild population size of *Ae*. *aegypti* in Tubiacanga was estimated by conventional models such as the Lincoln-Petersen index, which can be defined as $N_{L}=\frac{M\;n}{m}$, where *M* is the number of released females; *n* is the total number of females captured; and *m* is the number of marked individuals captured. Moreover, we also used the Fisher-Ford model, which takes into account mortality rate, which was directly estimated in the same MRR experiment, given by the function $N_{t}=\frac{R_{t}M\;\phi^{t}}{r_{t}}$, where *ϕ* is the PDS and *t* is the number of days elapsed after releases [27].

### Estimating the population size of wild mosquitoes using average-difference model

The population size of wild *Ae*. *aegypti* was estimated using an average-difference model based on *Wolbachia* releases on North Queensland, Australia. Population size was estimated by the increase of *Ae*. *aegypti* captures on BGS-Traps in the district in which *Wolbachia* was released in comparison with collections on a control site, in 2 weeks before and 2 weeks after releases [37]. We performed similar analysis in Rio, using the district of Jurujuba as a control site, where conditions for mosquito collections was similar to Tubiacanga. For comparing the control site with Tubiacanga we used the expanded data analysis to 4 weeks before and 4 weeks after releases.

### Ethics Statement

Mosquito releases in Tubiacanga were authorized by CONEP (CAAE 02524513.0.1001.0008), the National Research Council. The release of mosquitoes does not involve directly endangered or protected species and, from our experience, it does not have any significant impact on endangered or protected species.

## Results

### BGS Trap Collections

**Fig 1:**
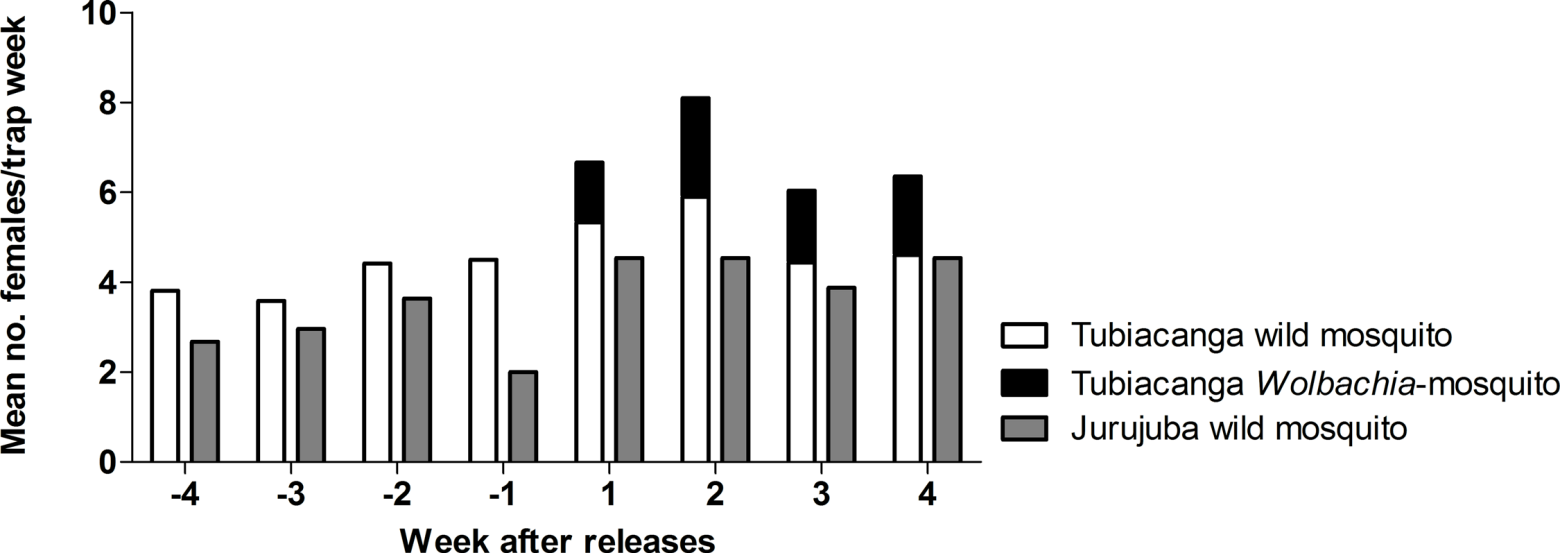
BG trap collections in field. Mean captures of female *Ae*. *aegypti* per BGS trap/week at the release site Tubiacanga and for the control area Jurujuba, for the periods of four weeks before and four weeks after the releases started. Mosquito captures increased after releases in Tubiacanga.

*Ae*. *aegypti* density was weekly monitored in Tubiacanga (release area) and Jurujuba (control area) before and after releasing *Wolbachia*-carrying mosquitoes (Fig 1). After releases started, the number of captured females increased substantially. In the second week of releases, the number of *Ae*. *aegypti* females captured was twice the average capture number from the pre-release period.

Daily samples were taken in the first two weeks after starting releases in Tubiacanga (Table 1). The capture rates were 1.7% (n = 40) on week 1 and 2.5% (n = 59) on week 2. The number of wild females was higher than the *Wolbachia*-carrying mosquitoes in all recapture days. Days 7 and 14 were release days and were removed from the analysis to avoid biases because there were no reliable methods to disentangle those released from the ones released a week earlier. During the releases, males were captured in lower numbers than females in BGS trap: 93 males in week 1 (among them, 23 were *Wolbachia*-infected) and 94 in week 2 (23 *Wolbachia*-infected).

**Table 1:** Daily capture rates after mosquito releases in Tubiacanga.

	Daily captures after release	
**Week 1**	*Wolbachia*-carrying mosquito	Wild mosquito
**Day 1**	12	42
**Day 2**	3	21
**Day 3**	8	16
**Day 4**	12	27
**Day 5**	3	28
**Day 6**	2	26
**Total**	40	160
**Per BG trap (total/number of traps)**	1.3	5.3
**Capture rate (total/number of released)**	1.7%	-
**Week 2**		
**Day 8**	12	47
**Day 9**	8	29
**Day 10**	17	35
**Day 11**	7	35
**Day 12**	10	33
**Day 13**	5	24
**Total**	59	203
**Per BG trap (total/number of traps)**	2.0	6.8
**Capture rate (total/number of released)**	2.5%	

### Estimation of daily survival of Wolbachia-carrying mosquitoes in field

Considering daily collections in the first two weeks of release, the estimation for the probability of daily survival (PDS) for *Wolbachia*-carrying mosquitoes in Tubiacanga was 0.82 in week 1, and 0.89 in week 2 (Table 2).

**Table 2:** Daily survival rate of *Wolbachia*-carrying females under field conditions.

		*Wolbachia*-carrying mosquito	Logarithm of counts		
**Week 1**	Day 1	12	2.56	Slope	-0.20
	Day 2	3	1.39	**exp(slope)**	**0.82**
	Day 3	8	2.20		
	Day 4	12	2.56		
	Day 5	3	1.39		
	Day 6	2	1.10		
**Week 2**	Day 8	12	2.56	Slope	-0.12
	Day 9	8	2.20	**exp(slope)**	**0.89**
	Day 10	17	2.89		
	Day 11	7	2.08		
	Day 12	10	2.40		
	Day 13	5	1.79		

### Estimating the population size of wild mosquitoes based on Wolbachia releases in the field: average-difference model

Using average-difference model we estimate the population size based on changes on BGS capture, 2 weeks before and 2 weeks after the releases (Fig 1). In this method, we calculated the mean number of wild females before releases as 4.5 females/trap. A significant increase was observed after releases started, since this ratio augmented to 6.7 and 8.1 females/trap in weeks 1 and 2, respectively (Table 3). The density of wild population was estimated at 70 and 55 females per trap (wk1 and wk2, respectably).

**Table 3:** Population size estimates using average-difference model.

Variable	Samples calculation (1/2 wk)
1) Mean no. of wild female *Ae*. *aegypti* per BGS trap, before release (95% CI) (-1/-2 wk)	4.5/4.5(1.3–7.5/ 2.7–6.3)
2) Mean no. of female *Ae*. *aegypti* per BGS trap after release of *Wolbachia*- infected mosquitoes (95% CI)	6.7/8.1(4.3–9.9/ 5.5–11.4)
3) Increase (mean ratio) in BGS collections of female due to released mosquitoes	2.2/3.6
4) Ratio of wild to released mosquitoes in BGS collections	2.0/1.3
5) Estimated no. of wild mosquitoes per trap = no. of released mosquitoes/trap x ratio of wild to released mosquitoes*	70/ 55
Estimated no. of wild mosquitoes per premise = no. of released mosquitoes/trap x ratio of wild to released mosquitoes x no. of traps / no. of premises	2.4/ 1.9
Estimated no. of wild mosquitoes per area (m^2^) = no. of released mosquitoes/trap x ratio of wild to released mosquitoes x no. of traps / total area	0.024/ 0.019

* The numbers of released mosquitoes/trap was estimated based on a mean of survival mosquitoes in field considering a daily survival of 0.8 (see more details on a S1 Table).

### Estimating the population size of wild mosquitoes based on Wolbachia releases in the field: using MRR models

We used MRR models such as the Lincoln-Petersen and Fisher and Ford, to estimate the population size on a daily basis using *Wolbachia* as a marker (Table 4). The total number estimated by the average-difference method is shown for comparison purposes. The highest numbers were estimated from Lincoln-Petersen model and analysis using the average-difference model resulted in lowest abundance quantities.

**Table 4:** Wild population size (with confidence intervals) of *Ae*. *aegypti*.

		Fisher-Ford	Lincoln-Petersen	Average-difference
**Week 1**	Day 1	6,303	7,690	-
	Day 2	8,589	12,788	-
	Day 3	2,418	4,392	-
	Day 4	2,259	5,008	-
	Day 5	6,233	16,856	-
	Day 6	6,342	20,925	-
	Mean	5,357 (3,355–7,360)	11,276 (6,365–16,188)	2,100 (634–3,565)
	Per premise	6.2 (3.9–8.5)	13.0 (7.3–18.6)	2.4 (0.7–4.1)
**Week 2**	Day 8	7,641	8,585	-
	Day 9	6,140	7,750	-
	Day 10	3,279	4,650	-
	Day 11	6,567	10,463	-
	Day 12	4,015	7,186	-
	Day 13	4,818	9,688	-
	Mean	5,410 (4,086–6,735)	8,053 (6,406–9,700)	1,650 (987–2,313)
	Per premise	6.2 (4.7–7.8)	9.3 (7.3–11.2)	1.9 (1.1–2.7)

## Discussion

The endosymbiont bacterium *Wolbachia* has been deployed in field trials as a novel intervention aiming to reduce arboviruses transmission. *Wolbachia*-infected *Ae*. *aegypti* mosquitoes are established in areas of North Queensland, Australia and Vietnam and ongoing releases are taking place in Brazil, Colombia and Indonesia [36, 45, 46]. One of the milestones to obtain a successful invasion of *Wolbachia* into the local population is to release a sufficient number of mosquitoes which exceeds the threshold invasion [38]. Such threshold for invasion highlights the need for reliable estimates on the population size of wild mosquitoes in the target area. Additionally, invasion is dependent on releasing a mosquito population not only fit to survive in the natural environment but also with a strong cytoplasmic incompatibility, a crucial mechanism to facilitate *Wolbachia* spread [35]. Using an MRR approach in which mosquitoes are individually screened for *Wolbachia* presence, we are able to estimate significant aspects of vector biology by performing daily collections of *Wolbachia*-infected *Ae*. *aegypti* mosquitoes released once a week. Herein, we provide estimates of two critical parameters for invasion success: the population size of wild mosquitoes and the probability of daily survival of *Wolbachia*-carrying mosquitoes in the field.

Ritchie et al. (2013) [37] and Nguyen et al. (2015) [47] took advantage of the *Wolbachia* releases to estimate wild *Ae*. *aegypti* density. However, in their work, the bacteria was not used as an individual marker in the field. Using the average-difference model, they assumed the increase in the capture rates after releases was due to the addition of *Wolbachia*-infected *Ae*. *aegypti* through releases. Using data from the first *Wolbachia* release in Latin America, we proposed individual *Wolbachia* screening to use not only the average-difference model but also the Lincoln-Petersen and Fisher-Ford models, and thus compared population size estimates among methods. Different from our results, Ritchie et al. (2013) and Nguyen et al. (2015) showed that released mosquitoes were more abundant than the wild ones, suggesting low vector infestation in release areas. For example, in two sites from North Queensland, a ratio of 1:1.2–1.7 (wild:*w*Mel) was observed during the first two weeks of *w*Mel releases [37]. In Vietnan, a ratio of 1:2–4 was observed during the *w*MelPop releases. Our results in Rio de Janeiro/Brazil using the same average-difference model showed a ratio of 1: 0.5–0.7, which could hinder *Wolbachia* invasion due to high density of native mosquitoes. Thus, a successful invasion may require the period for *Wolbachia* releases to last longer or/and an increase in the number of released mosquitoes.

Our estimates of BGS trapping efficiency for female *Wolbachia*-carrying mosquito (ranging from 1.7 to 2.5%) were lower when compared to other MRR experiments in Rio de Janeiro. Usually the recapture rates ranged from 7 to 15% [15, 29, 48]. Ritchie et al 2013 [37] in North Queensland had sampling rates of 5 to 10% after releasing *Wolbachia*-carrying mosquitoes. Our lower recapture rate might be explained by the limited number of BGS traps installed, i.e., roughly, we had one BG every 30 houses. Probably, recapture rates increase if additional collecting methods such as backpack aspirator are used [15].

Mosquito population in Rio de Janeiro presents strong variation over short periods of time, eventually doubling its recapture rate on an interval of few weeks [49]. Under this scenario, estimates on *Ae*. *aegypti* population size using the average-difference model may have limited reliability because a fraction of the increase in collections after *Wolbachia* releases could be due to a natural fluctuation of mosquito population (see Fig 1). In this case, mosquito counts might not be accurate, because the excess numbers are considered to be all *Wolbachia*-mosquitoes.

We estimated the PDS of *Wolbachia*-infected mosquitoes under field conditions, which ranged from 0.82 to 0.89 in the first two weeks after releases in Tubiacanga. This is the first estimation of daily survival of *Wolbachia Ae*. *aegypti* mosquitoes in the field using methodology from MRR literature. Overall, it seems the *w*Mel strain did not affect significantly the daily survival of *Ae*. *aegypti* [37], since the PDS values observed are similar or even higher than PDS values found for wild mosquitoes in previous studies [15, 28, 50]. In particular, Maciel-de-Freitas et al. (2007) [15] found a PDS ranging from 0.71 to 0.75 in Tubiacanga during dry and wet seasons of 2007. Other studies in Rio de Janeiro raised the hypothesis that *Ae*. *aegypti* PDS might depend on the urban landscape. For instance, a PDS of ~0.93 was observed in a highly dense typical Brazilian slum, whereas in suburban districts, PDS ranged from 0.73 to 0.89 [48]. Finally, in a sparsely populated high-income neighborhood, we observed the lowest PDS of Rio, varying from 0.61 to 0.70 [15, 39, 48, 51]. Under such scenarios the effect of urban landscape on mosquito survival is potentially due to the availability of human hosts and breeding sites. Maciel-de-Freitas et al. (2007b) [52] observed a tendency towards higher *Ae*. *aegypti* survival in areas with a high human density. In crowded districts such as slums, mosquitoes would not present a long flight to find host or breeding sites, reducing the odds of mortality due to harsh environmental factors, insecticide use or even by the defensive host behavior [53].

Given the low capture rates in the field, the exponential model is used extensively for estimating the probability of daily survival. Analysis under the exponential model does not consider the number of individuals captured to be removed from environment due to daily collections, which might impact subsequent collections. A possible approach for estimation under such conditions is to use the method proposed by Buonaccorsi et al (2003) [54].

By individually screening *Ae*. *aegypti* females for *Wolbachia*, we were able to estimate the population size of wild mosquitoes using three different models. MRR models provided higher values of population size than the average-difference model. Probably, the difference is because the average-difference model misdetected some wild mosquitoes as the *Wolbachia* ones, contributing to underestimate the wild population size (calculated as 2,100 week 1 and 1,650 week 2). This occurs due to large oscillation in number of wild mosquitoes between weeks in Rio de Janeiro. Therefore, MRR models under individual screening by qPCR give us more accurate quantities because in this case, we know precisely which mosquitoes had *Wolbachia*. Due to high mortality rates, Lincoln-Petersen results possibly overestimated population size (estimated as 11,276 week 1 and 8,053 week 2), since the model does not consider mortality rate in released mosquitoes. Finally, the index from Fisher-Ford model seems to be a reliable estimator of population size (5,357 week 1 and 5,410 week 2), since the model considers mortality rate during the experiment studies.

Our MRR results suggest that fitness of *Wolbachia*-infected mosquitoes in the field is high enough to promote invasion. By screening mosquitoes individually and using this information to generate quantities to be applied to MRR models, we avoided inaccurate estimations due to fluctuation in the average number of mosquitoes per week. Such technique is deemed highly important to determining in future release sites the minimum number of released individuals, by taking into account the wild population size in order to achieve a sustainable *Wolbachia* invasion over time.

## Supporting Information

S1 Table Estimating the number of *Wolbachia*-carrying *Ae*. *aegytpi* females in field, based on a daily survival of 0.8. (DOCX)
