# Supplementary material for: Using Wolbachia Releases to Estimate Aedes aegypti (Diptera: Culicidae) Population Size and Survival
Source: PLoS One. 2016 Aug 1;11(8):e0160196. doi: 10.1371/journal.pone.0160196 (PMC4968812; doi:10.1371/journal.pone.0160196)
Supplement: S1 Table — (DOCX) [file pone.0160196.s001.docx]

|  |  | **Total no. of *Wolbachia-*carrying mosquito in field** | ***Wolbachia*-carrying mosquito/trap** |
| --- | --- | --- | --- |
| **Release 1** |  | 2,350 (release) |  |
|  | Day 1 | 1,860 | 62 |
|  | Day 2 | 1,488 | 50 |
|  | Day 3 | 1,190 | 40 |
|  | Day 4 | 952 | 32 |
| **Week 1** | Day 5 | 762 | 25 |
|  | Day 6 | 609 | 20 |
|  | Day 7 | 488 | 16 |
|  | **Mean** | **1,050** | **35*** |
| **Release 2** |  | 2,350 + 488  (release + survival of week 1) |  |
|  | Day 8 | 2,250 | 75 |
|  | Day 9 | 1,800 | 60 |
|  | Day 10 | 1,440 | 48 |
|  | Day 11 | 1,152 | 38 |
| **Week 2** | Day 12 | 922 | 31 |
|  | Day 13 | 737 | 25 |
|  | Day 14 | 590 | 20 |
|  | **Mean** | **1,270** | **42*** |
